# Novel functions for the transcription factor E2F4 in development and disease

**DOI:** 10.1080/15384101.2016.1234551

**Published:** 2016-10-18

**Authors:** Jenny Hsu, Julien Sage

**Affiliations:** Departments of Pediatrics and Genetics, Stanford University, Stanford, CA, USA

**Keywords:** cancer, cell cycle, development, differentiation, E2F, E2F4, RB, regeneration, stem cells

## Abstract

The E2F family of transcription factors is a key determinant of cell proliferation in response to extra- and intra-cellular signals. Within this family, E2F4 is a transcriptional repressor whose activity is critical to engage and maintain cell cycle arrest in G0/G1 in conjunction with members of the retinoblastoma (RB) family. However, recent observations challenge this paradigm and indicate that E2F4 has a multitude of functions in cells besides this cell cycle regulatory role, including in embryonic and adult stem cells, during regenerative processes, and in cancer. Some of these new functions are independent of the RB family and involve direct activation of target genes. Here we review the canonical functions of E2F4 and discuss recent evidence expanding the role of this transcription factor, with a focus on cell fate decisions in tissue homeostasis and regeneration.

## Introduction

**Figure 1.**
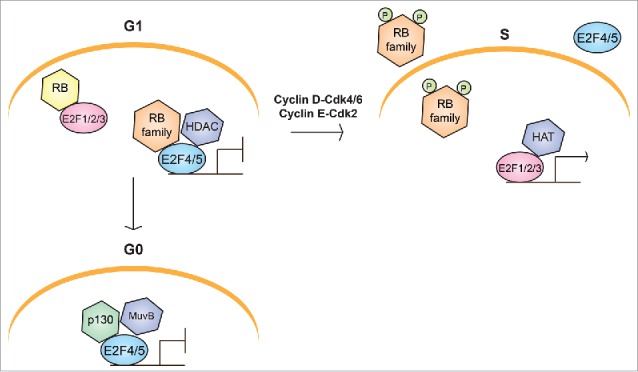
Schematic representation of the canonical RB/E2F pathway in cell cycle progression. In G1, cells can either enter S phase or exit the cell cycle into G0. Entry into S phase requires activation of transcriptional programs controlled by E2F activity. Binding of repressive complexes involving RB and E2F family members to the promoters of cell cycle genes silences their transcription in both G0 and G1. Repressive RB/E2F complexes consist of an RB family member, either E2F4 or E2F5, and additional chromatin modification and remodeling factors, including histone deacetylases (HDACs). In G0, repressive complexes generally contain p130 and the MuvB core complex (DREAM complex), whereas p107 predominates in G1. When cells enter S phase, Cyclin-CDK activity is upregulated and phosphorylates RB family proteins, promoting the dissociation of repressive RB/E2F complexes, and releasing “activator” E2Fs to upregulate the expression of cell cycle genes with histone acetyltransferases (HATs) and other chromatin-modifying factors.

The balance and function of diverse cell types in embryonic and adult tissues universally depend upon the ability of precursor cells to proliferate, commit to a differentiation program, and withdraw from the cell cycle. The molecular mechanisms that regulate and link these processes remain incompletely understood, but accumulating evidence in the past 3 decades has demonstrated a central role for the RB/E2F pathway (Fig. 1).^1-3^

E2F4 is a critical molecule in the RB/E2F pathway. Although E2F4 has been extensively studied as a repressor of cell cycle genes, here we present the novel perspective that E2F4 regulates diverse gene expression programs in cell fate decisions. We discuss possible mechanisms that support these new roles, as well as the implications of these roles for disease research, including regenerative medicine and cancer. Note that although E2F5 is structurally very similar and may function in similar contexts, we focus this review on E2F4 because it is highly conserved across evolution and featured in many recent studies.

### The canonical model of E2F4

**Figure 2.**
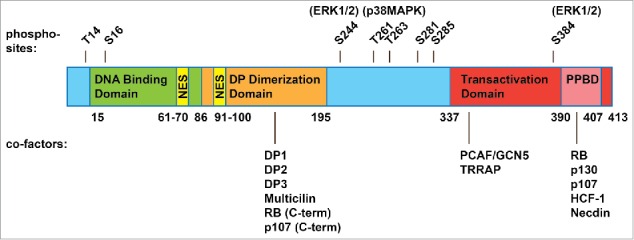
Structure of human E2F4. E2F4 is 413 amino acids long and contains a DNA binding domain (15-86), a dimerization domain that allows it to form heterodimers with a DP family protein (86-195), a transactivation domain (337-413), and within this, a pocket protein binding domain (PPBD, 390-407) that allows interactions with the RB family proteins. E2F4 shares these domains with the “activator” E2Fs and with E2F5. Yet unlike the “activator” E2Fs, E2F4 lacks a nuclear localization signal and shares with E2F5 a bipartite nuclear export signal (61-70, 91-100). E2F4 is thought to rely on the RB family proteins for nuclear localization, although post-translational modifications (such as phosphorylation sites, shown above with candidate kinases) and additional cofactors (shown below, with their interaction sites) may regulate E2F4 activity and cellular localization as well.

E2F4 belongs to the E2F family of transcription factors – 7 “classical” E2Fs (E2F1, E2F2, E2F3a, E2F3b, E2F4, E2F5, E2F6) that bind DNA with an essential dimerization partner protein (DP1-4); as well as 2 “atypical” E2Fs (E2F7 and E2F8) that function in a DP-independent manner.^4,5^ The classical E2Fs, with the exception of E2F6, physically interact with the RB family proteins (RB, p130, and p107) at the transactivation domain, and in some cases make contacts with the C-terminus of RB and p107 at the DP dimerization domain (Fig. 2).^6,7^ E2F4 associates with all 3 RB family proteins under physiological conditions, while E2F1-3 preferentially associate with RB, and E2F5 preferentially associates with p130.^8,9^ Interactions with the RB family at the transactivation domain prevent the recruitment of transcriptional machinery, inhibiting E2F activity. These tightly regulated interactions ensure that cell cycle genes are expressed at the appropriate cell cycle stages (Fig. 1).

Although E2F4 and E2F5 have a transcriptional activation domain, they are less important for gene activation in the canonical model of cell cycle progression. This is because they both lack a nuclear localization signal and are thought to rely on the RB family proteins for their nuclear translocation (Fig. 2), and to be sequestered in the cytoplasm in cycling cells (Fig. 1). Specifically, E2F4 export is mediated by 2 nuclear export signals (NES) that are recognized by the nuclear export receptor CRM1 (Fig. 2). Forced expression of CRM1 can prevent p16^INK4a^-induced cell cycle arrest in G1, an E2F4/5- dependent process.^10^ Thus, E2F1-3 are classically categorized as the “activator E2Fs,” responsible for triggering cell cycle entry, while E2F4-5 are “repressors” that prevent uncontrolled proliferation.^8,9^

E2F4 can also function in non-cycling cells as part of the DREAM complex, which generally consists of DP1, RBL2 (p130), E2F4, and the MuvB core proteins (RBBP4, LIN9, LIN37, LIN52, LIN54) (Fig. 1). p107, but not RB, can substitute for p130 in this complex. This complex is conserved in flies (dREAM) and presumably in *C. elegans* (DRM).^11-13^ Repression of cell cycle genes in G0 (quiescence, senescence, and differentiated states) may be achieved by recruiting chromatin-modifying factors such as HDAC1 and Sin3B, although how these factors interact with RB or the DREAM complex is still not fully understood.^14^ In general, the relative contributions of DREAM-bound E2F4 versus RB-bound E2F4 complexes in establishing and maintaining quiescence remain unclear.

A deeper look at the literature, however, challenges the canonical role of E2F4 as a repressor of cell cycle genes. Indeed, E2F4 is not required for G0/G1 arrest in all cell types. For instance, *E2f4*^*−/−*^;*E2f5*^*−/−*^ mouse embryonic fibroblasts can still undergo cell cycle arrest following serum deprivation,^15^ and E2F4 is only peripherally involved in the G1/S checkpoint in non-mammalian systems (see below). Here we will examine the non-canonical roles of E2F4 and discuss the importance of these roles for disease research and stem cell biology.

### The role of E2F4 in the development of different model organisms

E2F4 is highly conserved, and in some organisms represents the only E2F homolog: although ancestral E2F activity consists of one member each of E2F1/2/3/6, E2F4/5, and E2F7/8, many organisms do not have an E2F7/8 homolog and/or an activating E2F.^16^ While this observation highlights the importance of E2F4, loss of E2F4 function in model organisms does not negatively impact viability per se, but rather the development of diverse tissue types.

**Figure 3.**
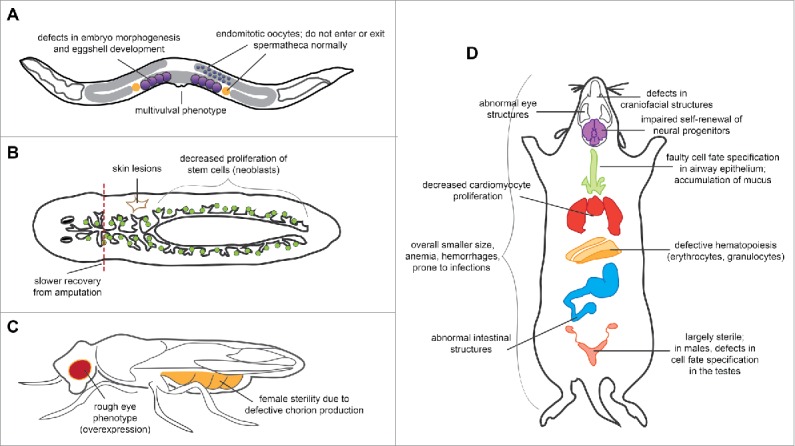
Summary of the developmental phenotypes associated with loss of E2F4 function in worms, flies and mice. Loss of E2F4 results in defects in multiple tissues of (A) *C. elegans*, (B) *S. mediterranea*, (C) *D. melanogaster*, and (D) *M. musculus* (see text). A number of these phenotypes have been attributed to cell cycle-independent or RB family-independent changes in gene expression and cell fate specification, suggesting that E2F4 may play context-dependent roles.

***Caenorhabditis elegans**. C. elegans* have 2 E2F genes (*efl-1* and *efl-2*), one DP gene (*dpl-1*), and one RB gene (*lin-35*). EFL-1 resembles E2F4 and E2F5 most strongly in its DP dimerization domain and its lack of a nuclear localization signal. EFL-1, DPL-1, and LIN-35 can form a ternary complex,^17^ and genes commonly bound by the 3 proteins across all tissues are enriched for cell cycle-related processes. However, the individual proteins also have tissue-specific targets, and EFL-1 is also required to antagonize Ras signaling during vulval development,^17^ to establish polarity for proper morphogenesis in embryos,^18^ and to regulate X chromosome genes in germ cells (Fig. 3).^19^ Importantly, loss of EFL-1 does not affect cell cycle progression in all cell types.^17,20^ In addition, EFL-1 and DPL-1, but not LIN-35, function in the germline to upregulate genes involved in oogenesis and fertility.^21^ Thus, EFL-1 may repress cell cycle genes in *C. elegans*, but may also function as a general transcription factor outside the canonical RB/E2F pathway in the development of different tissues.

***Schmidtea mediterranea***. Planaria have the simplest version of the RB/E2F pathway: a single RB homolog (*Smed-Rb*) that resembles p130 and p107, a single DP (*Smed-Dp*), and a single E2F (*Smed-E2f4-1*). Although knockdown of Smed-E2F4-1 temporarily increases cell division, consistent with its repressor function, knockdown animals eventually show slower stem cell proliferation, as well as phenotypes indicative of stem cell loss (Fig. 3). Whether these phenotypes are due to changes in apoptosis, differentiation, or proliferation has not been studied, although Smed-Rb is primarily involved in self-renewal and Smed-E2F4-1 may share this role as well.^16^

***Drosophila melanogaster***. Flies have 2 E2F genes (*de2f1* and *de2f2*), one DP gene (*ddp*), and 2 RB-like genes (*rbf1* and *rbf2*). dE2F1 and dE2F2 function respectively as an activator and a repressor of transcription, and are considered the fly equivalents of E2F1 and E2F4. RBF1 interacts with both E2Fs, while RBF2 only interacts with dE2F2.^22^ Like in mammalian systems, dE2F1 and dE2F2 play antagonistic roles in cell cycle regulation, and co-expression of dE2F2 and RBF2 in vivo slows cell cycle progression.^22,23^ However, neither loss nor overexpression of dE2F2 alone has much effect on cell cycle stages or the expression of cell cycle genes. Instead, loss of dE2F2 can sometimes result in female sterility due to defects in chorion development,^24^ while overexpression of dE2F2 results in a rough eye phenotype (Fig. 3).^23^ Strikingly, loss of dE2F2 also leads to the de-repression of many genes involved in oogenesis, sex specification, and male courtship behavior.^12,25-27^ Additional assays in future fly studies may therefore reveal novel phenotypes in these areas.

***Mus musculus**. E2f4*^*−/−*^ mice are smaller than wild-type mice and have defects in multiple tissues and organs, including blood, bone, skin, intestinal tissue, visual system, and reproductive system (Fig. 3).^28-32^ The majority of *E2f4*^*−/−*^ mice die within the first few weeks of life from defects in craniofacial structure that increase their susceptibility to bacterial infections.^28^ In addition, *E2f4*^*−/−*^ mice are largely sterile, even when bred to normal mice.^28,29^

Although the mechanisms behind most of these defects are not well studied, evidence overwhelmingly suggests a context-dependent role for E2F4 in different cell types rather than a more general role in the cell cycle. For example, E2F4 has a cell cycle-independent role in regulating sonic hedgehog (Shh) in the ventral telencephalon, which controls the self-renewal of neural precursor cells.^30^ In addition, very recent studies show that E2F4 can upregulate genes involved in the development of cilia in multiciliated cells, in a complex with DP, Multicilin, and GEMC1.^33,34^ Consistently, loss of E2F4 prevents the appearance of ciliated cells in the airway epithelium^35^ and the male reproductive system,^32^ ultimately leading to the bacterial infections and sterility in *E2f4*^*−/−*^ mice. Interestingly, loss of E2F4 also precedes the downregulation of genes involved in endocytosis and water channel transport in the testes.^32^ Exploring whether E2F4 directly activates these genes may provide further evidence for E2F4 as a general transcription factor rather than a repressor of cell cycle genes. As discussed above, the E2F4-containing complexes (with RB or DREAM, and possibly others) that mediate these different functions in vivo are still poorly understood.

### E2F4 in rapidly cycling cells and cancer

Although E2F4 is categorized as the major repressor of cell cycle progression, loss of E2F4 in the cycling stem and progenitor populations of multiple tissue types actually decreases proliferation and DNA replication. This phenomenon is best observed in intestinal tissue, as *E2f4*^*−/−*^ mice exhibit a reduced or absent crypt region and poorly developed villi (although it is possible these defects are secondary to developmental defects).^32^ Accordingly, knockdown of E2F4 in human intestinal epithelial cells (HIECs), a cell culture model of the intestinal crypt, leads to decreased proliferation and a downregulation of direct E2F targets.^36-38^ E2F4 even seems to supplant E2F1 as the primary activator E2F in this tissue type, as nuclear E2F4 is strongly expressed in the proliferative zone of the intestinal crypt, whereas E2F1 expression is diffuse and does not depend on the phases of the cell cycle.^36^ Similar pro-proliferative effects for E2F4 have also been observed in blood cells^39^ and in the developing epidermis.^40,41^ Whether E2F4 directly activates cell cycle genes in these contexts is not well characterized.

In cancers, E2F4 appears to act primarily as an oncogene, which is more consistent with its non-canonical role in pro-proliferative cells than with its canonical, repressive role in the cell cycle. Prostate tumors^42^ and breast cancers^43,44^ express E2F4 at higher levels than surrounding normal tissue, and its nuclear expression in breast cancer strongly correlates with poor prognosis. High levels of E2F4 are also present in a mouse model of skin cancer, and overexpression of E2F4 in epidermis, particularly in conjunction with DP1, results in skin tumors.^40^ It is tempting to speculate that E2F4 might drive aberrant cell cycle progression in these contexts by switching from a repressor to an activator at the promoters of cell cycle genes. However, E2F4 can directly repress apoptotic genes,^45^ and therefore E2F4 might also promote tumor growth by protecting cancer cells more efficiently against cell death.

Indeed, another fascinating pro-tumorigenic role of E2F4 is that it is co-opted by some tumors to escape cell death following DNA damage. Cancer cells generally lack a G1/S checkpoint and are unable to undergo G1 arrest during DNA damage. Instead, compromised cells arrest in G2 by upregulating E2F4 and p130, and turning on a mechanism that involves repression of G2/M genes by E2F4-p130.^46-48^ In some tumors, E2F4-p130 also binds to and represses genes involved in DNA damage repair, such as RAD51 and BRCA1 (in homologous recombination)^46,49,50^ and XPC (in nucleotide excision repair),^51^ possibly allowing genomically unstable cells to survive. Knockdown of E2F4 in these contexts prevents G2 arrest and sensitizes cancer cells to irradiation-induced apoptosis.

### Mechanisms of E2F4 activity

The versatility of E2F4 is intriguing, but makes sense given that E2F4 is widely expressed and binds diverse targets, including enhancer regions and regions without an E2F consensus sequence.^52^ Indeed, the role of E2F4 in the differentiation of multiple tissues has been attributed to its direct regulation of non-cell cycle genes such as *PPARG* (during adipogenesis),^53^ and *deup1* (in centriole amplification and cilia development).^33,34^ Furthermore, E2F4 may guide the differentiation of pluripotent stem cells by directly repressing pluripotency factors such as *Sox2* in conjunction with RB and p130.^54,55^ Notably, while DREAM represses targets in G0 that are involved in centrosome function, mRNA processing, and metabolism,^56^ it also represses developmental genes in proliferating cells to promote cell cycle progression in flies,^57^ and to allow expansion of precursor cells in mammalian bone development.^58^ The repression of diverse targets in different cell cycle phases suggests a more general role for E2F4 in cell fate specification rather than simply as a regulator of the G0 and G1/S phases. As our knowledge of E2F4 targets is still limited to ChIP-Seq data from a handful of studies^33,52,59^ and from well established ENCODE lines, it will be important to obtain ChIP-Seq datasets from additional cell types (e.g. differentiating adipocytes) to test this idea.

In addition, what allows E2F4 to regulate different gene programs in different cell types is not well understood. *In vitro* and ChIP experiments have shown that cofactors may influence the binding motif preferences of the individual E2Fs, directing E2F4 to different binding sites^52,60^ and may also determine whether E2F4 functions as a repressor or an activator. For instance, while the nuclear localization of E2F4 in G0 and G1 depends on p130 and p107, these interactions inhibit the E2F4 transactivation domain, allowing the formation of a purely repressive complex (Fig. 2). In contexts where the RB family proteins are inactive, E2F4 might function primarily as an activator, likely with a co-activator that allows it to translocate into the nucleus or increases its nuclear retention (for example by masking the NES). Consistently, E2F4 drives proliferation in stem and progenitor cells and in cancer cells, which are rapidly cycling and in which the RB family proteins are inactive or mutated. In one striking example, the proliferative cells of the intestinal crypt express cytoplasmic, inactive RB family proteins, while E2F4 is largely nuclear.^36^ Similarly, in fetal liver where E2F4 drives erythropoiesis, the majority of E2F4 binding activity consists of a “free” form of E2F4 that does not associate with the RB family proteins, with other E2Fs expressed at a low level or not at all.^39^

Thus far, very few RB family-independent cofactors and their contributions to E2F4 activity have been defined. These include HCF-1, in early G1,^61^ and necdin, with which E2F4 represses target genes during adipocyte differentiation (Fig. 2).^53^ Alternatively, E2F4 can also inhibit adipocyte differentiation in an RB family-independent manner, although the cofactors involved in this process are not known.^62^ Even less is known about cofactors that allow E2F4 to localize to the nucleus and function as an activator in RB family-inactive, rapidly cycling cells. Candidates include HCF-1, which also associates with E2F4 in S phase;^61^ GCN5 and TRRAP, which recruit histone acetyltransferases to drive E2F-dependent transactivation;^63^ and Multicilin and GEMC1, which guide E2F4 to activate genes required for centriole biogenesis. (Fig. 2)^33,34^ In addition, DP-3 and DP-2 can both promote nuclear localization of E2F4 and E2F5 in an RB family-independent manner, as well as cell cycle progression.^64-66^ DP-2 associates with E2F4 in embryonic stem cells,^67^ and perhaps E2F4 prefers to heterodimerize with DP-2 or DP-3 (instead of DP-1) in cycling cells (Fig. 2).

Finally, other mechanisms for RB family-independent transport may include association with subcellular structures, such as kinetochores (as in cardiomyocyte proliferation),^68^ and differential phosphorylation. For instance, ERK1/2-mediated phosphorylation of E2F4 on serines S244 and S384 promotes its nuclear localization in proliferating intestinal cells.^38^ Phosphorylation of E2F4 on threonines T261 and T263 by p38^MAPK^ allows it to bind and activate cell cycle genes during cell cycle re-entry of neurons (Fig. 2).^69^ Improvements in affinity purification and mass spectrometry might allow more cofactors and post-translational modifications to be identified in different tissue-specific contexts.

## Conclusions

**Figure 4.**
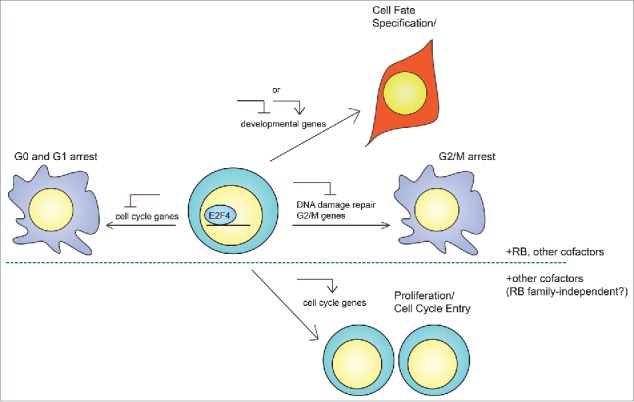
Potential mechanisms of action of E2F4 in stem cells. In addition to its canonical role as a repressor of cell cycle progression in G0/G1, E2F4 may have at least 3 different functions as a general transcription factor in adult stem cells and embryonic stem cells (ESCs). First, E2F4 may regulate developmental genes to establish cell fate during differentiation, either as a repressor in conjunction with RB to prevent the expression of aberrant transcripts, or as an activator to directly drive cellular differentiation. Second, rapidly cycling cells largely lack a G1/S checkpoint and may utilize E2F4 to undergo arrest in G2 in response to cellular stress, during which E2F4 represses genes involved in G2/M progression and DNA damage repair. Third, E2F4 may switch from a repressor to an activator of cell cycle genes to support heightened metabolic requirements. While the first 2 cases may involve formation of complexes containing E2F4 and a RB family member, the third may not require RB family proteins, which would inhibit E2F4 transcriptional activity. In addition, all 3 cases may involve additional cofactors that facilitate E2F4 translocation into the nucleus and binding to target genes.

A better understanding of the non-canonical roles and mechanisms for E2F4 will greatly benefit many fields, including stem cell and cancer biology. First, studying the repressor function of E2F4 in terminally differentiated cell types, and identifying additional non-canonical targets, may shed more light on the mechanisms that control cell cycle arrest and differentiation. In embryonic stem cells, for instance, RB mediates differentiation at least in part by direct repression of pluripotency genes.^54,55,70^ As E2F4 directly represses Sox2,^55^ it may also silence other pluripotency genes in conjunction with RB to establish proper cell fate (Fig. 4).

Second, the activator role of E2F4 in rapidly proliferating cell types might be important for improving the function of adult stem cells during regeneration. Indeed, E2F4 drives the proliferation of cardiomyocytes, which declines drastically during early embryonic development as nuclear E2F4 expression decreases. Importantly, the cell cycle re-entry of adult cardiomyocytes in both normal mice and in a mouse model of myocardial infarction requires an increase in nuclear E2F4.^68^ In addition, neuronal regeneration and recovery of mobility in zebrafish following spinal cord injury requires an increase in E2F4 activity.^71^ Understanding these roles might shed light on how to reverse the effects of age-related and neurodegenerative diseases.

Finally, the role of E2F4 in undifferentiated embryonic stem cells (ESCs) and cancer cells remains to be explored. As ESCs and cancer cells are similar in cell cycle structure, research on E2F4 in ESCs might inform research in cancer biology, and vice versa. Although *E2f4*^*−/−*^ ESCs grow normally in physiological conditions,^28,29^ E2F4 binds to ∼6000 promoters in ESCs, despite the absence of a G1/S checkpoint,^67^ and represents ∼95% of E2F DNA binding activity in ESCs.^28^ One idea is that E2F4 is poised to regulate the expression of developmental genes during differentiation (Fig. 4). Additionally, ESCs resemble cancer cells in their DNA damage response, and thus E2F4 may mediate G2/M arrest or repress DNA damage repair genes in both cell types through similar mechanisms (Fig. 4). Finally, an intriguing hypothesis is that E2F4 might directly control the cell cycle of ESCs by activating the expression of cell cycle genes in an RB family-independent manner (Fig. 4). Indeed, E2F4 co-binds promoters in ESCs with Myc, a strong transcriptional activator that is critical for ESC self-renewal and cell cycle entry.^67,72^ Thus, understanding the non-canonical functions of E2F4 will likely reveal novel insights into pluripotency and differentiation, which might in turn guide the development of strategies to block the expansion of cancer cells.

## References

[1] Chinnam M, Goodrich DW. RB1, development, and cancer. Curr Top Dev Biol 2011; 94:129-69; PMID:21295686; http://dx.doi.org/10.1016/B978-0-12-380916-2.00005-X 21295686 PMC3691055

[2] Conklin JF, Baker J, Sage J. The RB family is required for the self-renewal and survival of human embryonic stem cells. Nat Commun 2012; 3:1244; PMID:23212373; http://dx.doi.org/10.1038/ncomms2254 23212373

[3] Dyson NJ. RB1: a prototype tumor suppressor and an enigma. Genes Dev 2016; 30:1492-502; PMID:27401552; http://dx.doi.org/10.1101/gad.282145.116 27401552 PMC4949322

[4] Dimova DK, Dyson NJ. The E2F transcriptional network: old acquaintances with new faces. Oncogene 2005; 24:2810-26; PMID:15838517; http://dx.doi.org/10.1038/sj.onc.1208612 15838517

[5] Lammens T, Li J, Leone G, De Veylder L. Atypical E2Fs: new players in the E2F transcription factor family. Trends Cell Biol 2009; 19:111-8; PMID:19201609; http://dx.doi.org/10.1016/j.tcb.2009.01.002 19201609 PMC2808192

[6] Zhu L, Enders G, Lees JA, Beijersbergen RL, Bernards R, Harlow E. The pRB-related protein p107 contains two growth suppression domains: independent interactions with E2F and cyclin/cdk complexes. EMBO J 1995; 14:1904-13; PMID:7743997 7743997 10.1002/j.1460-2075.1995.tb07182.x PMC398289

[7] Rubin SM, Gall AL, Zheng N, Pavletich NP. Structure of the Rb C-terminal domain bound to E2F1-DP1: a mechanism for phosphorylation-induced E2F release. Cell 2005; 123:1093-106; PMID:16360038; http://dx.doi.org/10.1016/j.cell.2005.09.044 16360038

[8] Trimarchi JM, Lees JA. Sibling rivalry in the E2F family. Nat Rev Mol Cell Biol 2002; 3:11-20; PMID:11823794; http://dx.doi.org/10.1038/nrm714 11823794

[9] Bertoli C, Skotheim JM, de Bruin RA. Control of cell cycle transcription during G1 and S phases. Nat Rev Mol Cell Biol 2013; 14:518-28; PMID:23877564; http://dx.doi.org/10.1038/nrm3629 23877564 PMC4569015

[10] Gaubatz S, Lees JA, Lindeman GJ, Livingston DM. E2F4 is exported from the nucleus in a CRM1-dependent manner. Mol Cell Biol 2001; 21:1384-92; PMID:11158323; http://dx.doi.org/10.1128/MCB.21.4.1384-1392.2001 11158323 PMC99590

[11] Korenjak M, Taylor-Harding B, Binne UK, Satterlee JS, Stevaux O, Aasland R, White-Cooper H, Dyson N, Brehm A. Native E2F/RBF complexes contain Myb-interacting proteins and repress transcription of developmentally controlled E2F target genes. Cell 2004; 119:181-93; PMID:15479636; http://dx.doi.org/10.1016/j.cell.2004.09.034 15479636

[12] Lewis PW, Beall EL, Fleischer TC, Georlette D, Link AJ, Botchan MR. Identification of a Drosophila Myb-E2F2/RBF transcriptional repressor complex. Genes Dev 2004; 18:2929-40; PMID:15545624; http://dx.doi.org/10.1101/gad.1255204 15545624 PMC534653

[13] Harrison MM, Ceol CJ, Lu X, Horvitz HR. Some C. elegans class B synthetic multivulva proteins encode a conserved LIN-35 Rb-containing complex distinct from a NuRD-like complex. Proc Natl Acad Sci U S A 2006; 103:16782-7; PMID:17075059; http://dx.doi.org/10.1073/pnas.0608461103 17075059 PMC1636532

[14] Rayman JB, Takahashi Y, Indjeian VB, Dannenberg JH, Catchpole S, Watson RJ, te Riele H, Dynlacht BD. E2F mediates cell cycle-dependent transcriptional repression in vivo by recruitment of an HDAC1/mSin3B corepressor complex. Genes Dev 2002; 16:933-47; PMID:11959842; http://dx.doi.org/10.1101/gad.969202 11959842 PMC152357

[15] Gaubatz S, Lindeman GJ, Ishida S, Jakoi L, Nevins JR, Livingston DM, Rempel RE. E2F4 and E2F5 play an essential role in pocket protein-mediated G1 control. Mol Cell 2000; 6:729-35; PMID:11030352; http://dx.doi.org/10.1016/S1097-2765(00)00071-X 11030352

[16] Zhu SJ, Pearson BJ. The Retinoblastoma pathway regulates stem cell proliferation in freshwater planarians. Dev Biol 2013; 373:442-52; PMID:23123964; http://dx.doi.org/10.1016/j.ydbio.2012.10.025 23123964

[17] Ceol CJ, Horvitz HR. dpl-1 DP and efl-1 E2F act with lin-35 Rb to antagonize Ras signaling in C. elegans vulval development. Mol Cell 2001; 7:461-73; PMID:11463372; http://dx.doi.org/10.1016/S1097-2765(01)00194-0 11463372

[18] Page BD, Guedes S, Waring D, Priess JR. The C. elegans E2F- and DP-related proteins are required for embryonic asymmetry and negatively regulate Ras/MAPK signaling. Mol Cell 2001; 7:451-60; PMID:11463371; http://dx.doi.org/10.1016/S1097-2765(01)00193-9 11463371

[19] Tabuchi TM, Rechtsteiner A, Strome S, Hagstrom KA. Opposing activities of DRM and MES-4 tune gene expression and X-chromosome repression in Caenorhabditis elegans germ cells. G3 (Bethesda) 2014; 4:143-53; PMID:24281426; http://dx.doi.org/full_text 24281426 10.1534/g3.113.007849 PMC3887530

[20] Kudron M, Niu W, Lu Z, Wang G, Gerstein M, Snyder M, Reinke V. Tissue-specific direct targets of Caenorhabditis elegans Rb/E2F dictate distinct somatic and germline programs. Genome Biol 2013; 14:R5; PMID:23347407; http://dx.doi.org/10.1186/gb-2013-14-1-r5 23347407 PMC4053757

[21] Chi W, Reinke V. Promotion of oogenesis and embryogenesis in the C. elegans gonad by EFL-1/DPL-1 (E2F) does not require LIN-35 (pRB). Development 2006; 133:3147-57; PMID:16854972; http://dx.doi.org/10.1242/dev.02490 16854972

[22] Stevaux O, Dimova D, Frolov MV, Taylor-Harding B, Morris E, Dyson N. Distinct mechanisms of E2F regulation by Drosophila RBF1 and RBF2. EMBO J 2002; 21:4927-37; PMID:12234932; http://dx.doi.org/10.1093/emboj/cdf501 12234932 PMC126297

[23] Frolov MV, Huen DS, Stevaux O, Dimova D, Balczarek-Strang K, Elsdon M, Dyson NJ. Functional antagonism between E2F family members. Genes Dev 2001; 15:2146-60; PMID:11511545; http://dx.doi.org/10.1101/gad.903901 11511545 PMC312757

[24] Cayirlioglu P, Bonnette PC, Dickson MR, Duronio RJ. Drosophila E2f2 promotes the conversion from genomic DNA replication to gene amplification in ovarian follicle cells. Development 2001; 128:5085-98; PMID:11748144 11748144 10.1242/dev.128.24.5085

[25] Dimova DK, Stevaux O, Frolov MV, Dyson NJ. Cell cycle-dependent and cell cycle-independent control of transcription by the Drosophila E2F/RB pathway. Genes Dev 2003; 17:2308-20; PMID:12975318; http://dx.doi.org/10.1101/gad.1116703 12975318 PMC196467

[26] Georlette D, Ahn S, MacAlpine DM, Cheung E, Lewis PW, Beall EL, Bell SP, Speed T, Manak JR, Botchan MR. Genomic profiling and expression studies reveal both positive and negative activities for the Drosophila Myb MuvB/dREAM complex in proliferating cells. Genes Dev 2007; 21:2880-96; PMID:17978103; http://dx.doi.org/10.1101/gad.1600107 17978103 PMC2049191

[27] Stevaux O, Dimova DK, Ji JY, Moon NS, Frolov MV, Dyson NJ. Retinoblastoma family 2 is required in vivo for the tissue-specific repression of dE2F2 target genes. Cell Cycle 2005; 4:1272-80; PMID:16082225; http://dx.doi.org/10.4161/cc.4.9.1982 16082225

[28] Humbert PO, Rogers C, Ganiatsas S, Landsberg RL, Trimarchi JM, Dandapani S, Brugnara C, Erdman S, Schrenzel M, Bronson RT, et al. E2F4 is essential for normal erythrocyte maturation and neonatal viability. Mol Cell 2000; 6:281-91; PMID:10983976; http://dx.doi.org/10.1016/S1097-2765(00)00029-0 10983976

[29] Rempel RE, Saenz-Robles MT, Storms R, Morham S, Ishida S, Engel A, Jakoi L, Melhem MF, Pipas JM, Smith C, et al. Loss of E2F4 activity leads to abnormal development of multiple cellular lineages. Mol Cell 2000; 6:293-306; PMID:10983977; http://dx.doi.org/10.1016/S1097-2765(00)00030-7 10983977

[30] Ruzhynsky VA, McClellan KA, Vanderluit JL, Jeong Y, Furimsky M, Park DS, Epstein DJ, Wallace VA, Slack RS. Cell cycle regulator E2F4 is essential for the development of the ventral telencephalon. J Neurosci 2007; 27:5926-35; PMID:17537963; http://dx.doi.org/10.1523/JNEUROSCI.1538-07.2007 17537963 PMC6672261

[31] Ruzhynsky VA, Furimsky M, Park DS, Wallace VA, Slack RS. E2F4 is required for early eye patterning. Dev Neurosci 2009; 31:238-46; PMID:19325228; http://dx.doi.org/10.1159/000210186 19325228

[32] Danielian PS, Hess RA, Lees JA. E2f4 and E2f5 are essential for the development of the male reproductive system. Cell Cycle 2016; 15:250-60; PMID:26825228; http://dx.doi.org/10.1080/15384101.2015.1121350 26825228 PMC4825840

[33] Ma L, Quigley I, Omran H, Kintner C. Multicilin drives centriole biogenesis via E2f proteins. Genes Dev 2014; 28:1461-71; PMID:24934224; http://dx.doi.org/10.1101/gad.243832.114 24934224 PMC4083089

[34] Terre B, Piergiovanni G, Segura-Bayona S, Gil-Gomez G, Youssef SA, Attolini CS, Wilsch-Brauninger M, Jung C, Rojas AM, Marjanovic M, et al. GEMC1 is a critical regulator of multiciliated cell differentiation. EMBO J 2016; 35:942-60; PMID:26933123; http://dx.doi.org/10.15252/embj.201592821 26933123 PMC5207319

[35] Danielian PS, Bender Kim CF, Caron AM, Vasile E, Bronson RT, Lees JA. E2f4 is required for normal development of the airway epithelium. Dev Biol 2007; 305:564-76; PMID:17383628; http://dx.doi.org/10.1016/j.ydbio.2007.02.037 17383628 PMC1939821

[36] Deschenes C, Alvarez L, Lizotte ME, Vezina A, Rivard N. The nucleocytoplasmic shuttling of E2F4 is involved in the regulation of human intestinal epithelial cell proliferation and differentiation. J Cell Physiol 2004; 199:262-73; PMID:15040009; http://dx.doi.org/10.1002/jcp.10455 15040009

[37] Garneau H, Paquin MC, Carrier JC, Rivard N. E2F4 expression is required for cell cycle progression of normal intestinal crypt cells and colorectal cancer cells. J Cell Physiol 2009; 221:350-8; PMID:19562678; http://dx.doi.org/10.1002/jcp.21859 19562678

[38] Paquin MC, Cagnol S, Carrier JC, Leblanc C, Rivard N. ERK-associated changes in E2F4 phosphorylation, localization and transcriptional activity during mitogenic stimulation in human intestinal epithelial crypt cells. BMC Cell Biol 2013; 14:33; PMID:23919615; http://dx.doi.org/10.1186/1471-2121-14-33 23919615 PMC3750237

[39] Kinross KM, Clark AJ, Iazzolino RM, Humbert PO. E2f4 regulates fetal erythropoiesis through the promotion of cellular proliferation. Blood 2006; 108:886-95; PMID:16861343; http://dx.doi.org/10.1182/blood-2005-09-008656 16861343

[40] Wang D, Russell J, Xu H, Johnson DG. Deregulated expression of DP1 induces epidermal proliferation and enhances skin carcinogenesis. Mol Carcinog 2001; 31:90-100; PMID:11429786; http://dx.doi.org/10.1002/mc.1044 11429786

[41] Wang D, Russell JL, Johnson DG. E2F4 and E2F1 have similar proliferative properties but different apoptotic and oncogenic properties in vivo. Mol Cell Biol 2000; 20:3417-24; PMID:10779331; http://dx.doi.org/10.1128/MCB.20.10.3417-3424.2000 10779331 PMC85634

[42] Waghray A, Schober M, Feroze F, Yao F, Virgin J, Chen YQ. Identification of differentially expressed genes by serial analysis of gene expression in human prostate cancer. Cancer Res 2001; 61:4283-6; PMID:11358857 11358857

[43] Rakha EA, Armour JA, Pinder SE, Paish CE, Ellis IO. High-resolution analysis of 16q22.1 in breast carcinoma using DNA amplifiable probes (multiplex amplifiable probe hybridization technique) and immunohistochemistry. Int J Cancer 2005; 114:720-9; PMID:15609312; http://dx.doi.org/10.1002/ijc.20738 15609312

[44] Rakha EA, Pinder SE, Paish EC, Robertson JF, Ellis IO. Expression of E2F-4 in invasive breast carcinomas is associated with poor prognosis. J Pathol 2004; 203:754-61; PMID:15221934; http://dx.doi.org/10.1002/path.1573 15221934

[45] Dingar D, Konecny F, Zou J, Sun X, von Harsdorf R. Anti-apoptotic function of the E2F transcription factor 4 (E2F4)/p130, a member of retinoblastoma gene family in cardiac myocytes. J Mol Cell Cardiol 2012; 53:820-8; PMID:22985930; http://dx.doi.org/10.1016/j.yjmcc.2012.09.004 22985930

[46] Ren B, Cam H, Takahashi Y, Volkert T, Terragni J, Young RA, Dynlacht BD. E2F integrates cell cycle progression with DNA repair, replication, and G(2)/M checkpoints. Genes Dev 2002; 16:245-56; PMID:11799067; http://dx.doi.org/10.1101/gad.949802 11799067 PMC155321

[47] DuPree EL, Mazumder S, Almasan A. Genotoxic stress induces expression of E2F4, leading to its association with p130 in prostate carcinoma cells. Cancer Res 2004; 64:4390-3; PMID:15231644; http://dx.doi.org/10.1158/0008-5472.CAN-03-3695 15231644

[48] Crosby ME, Jacobberger J, Gupta D, Macklis RM, Almasan A. E2F4 regulates a stable G2 arrest response to genotoxic stress in prostate carcinoma. Oncogene 2007; 26:1897-909; PMID:17043659; http://dx.doi.org/10.1038/sj.onc.1209998 17043659 PMC2593901

[49] Bindra RS, Glazer PM. Repression of RAD51 gene expression by E2F4/p130 complexes in hypoxia. Oncogene 2007; 26:2048-57; PMID:17001309; http://dx.doi.org/10.1038/sj.onc.1210001 17001309

[50] Hegan DC, Lu Y, Stachelek GC, Crosby ME, Bindra RS, Glazer PM. Inhibition of poly(ADP-ribose) polymerase down-regulates BRCA1 and RAD51 in a pathway mediated by E2F4 and p130. Proc Natl Acad Sci U S A 2010; 107:2201-6; PMID:20133863; http://dx.doi.org/10.1073/pnas.0904783107 20133863 PMC2836641

[51] Dominguez-Brauer C, Chen YJ, Brauer PM, Pimkina J, Raychaudhuri P. ARF stimulates XPC to trigger nucleotide excision repair by regulating the repressor complex of E2F4. EMBO Rep 2009; 10:1036-42; PMID:19644500; http://dx.doi.org/10.1038/embor.2009.139 19644500 PMC2750060

[52] Lee BK, Bhinge AA, Iyer VR. Wide-ranging functions of E2F4 in transcriptional activation and repression revealed by genome-wide analysis. Nucleic Acids Res 2011; 39:3558-73; PMID:21247883; http://dx.doi.org/10.1093/nar/gkq1313 21247883 PMC3089461

[53] Tseng YH, Butte AJ, Kokkotou E, Yechoor VK, Taniguchi CM, Kriauciunas KM, Cypess AM, Niinobe M, Yoshikawa K, Patti ME, et al. Prediction of preadipocyte differentiation by gene expression reveals role of insulin receptor substrates and necdin. Nat Cell Biol 2005; 7:601-11; PMID:15895078; http://dx.doi.org/10.1038/ncb1259 15895078

[54] Kareta MS, Gorges LL, Hafeez S, Benayoun BA, Marro S, Zmoos AF, Cecchini MJ, Spacek D, Batista LF, O'Brien M, et al. Inhibition of pluripotency networks by the Rb tumor suppressor restricts reprogramming and tumorigenesis. Cell Stem Cell 2015; 16:39-50; PMID:25467916; http://dx.doi.org/10.1016/j.stem.2014.10.019 25467916 PMC4389904

[55] Li H, Collado M, Villasante A, Matheu A, Lynch CJ, Canamero M, Rizzoti K, Carneiro C, Martinez G, Vidal A, et al. p27(Kip1) directly represses Sox2 during embryonic stem cell differentiation. Cell Stem Cell 2012; 11:845-52; PMID:23217425; http://dx.doi.org/10.1016/j.stem.2012.09.014 23217425 PMC3549496

[56] Cam H, Balciunaite E, Blais A, Spektor A, Scarpulla RC, Young R, Kluger Y, Dynlacht BD. A common set of gene regulatory networks links metabolism and growth inhibition. Mol Cell 2004; 16:399-411; PMID:15525513; http://dx.doi.org/10.1016/j.molcel.2004.09.037 15525513

[57] Lee H, Ragusano L, Martinez A, Gill J, Dimova DK. A dual role for the dREAM/MMB complex in the regulation of differentiation-specific E2F/RB target genes. Mol Cell Biol 2012; 32:2110-20; PMID:22451490; http://dx.doi.org/10.1128/MCB.06314-11 22451490 PMC3372228

[58] Flowers S, Beck GR Jr, Moran E. Tissue-specific gene targeting by the multiprotein mammalian DREAM complex. J Biol Chem 2011; 286:27867-71; PMID:21685383; http://dx.doi.org/10.1074/jbc.C111.255091 21685383 PMC3151031

[59] Beck S, Lee BK, Rhee C, Song J, Woo AJ, Kim J. CpG island-mediated global gene regulatory modes in mouse embryonic stem cells. Nat Commun 2014; 5:5490; PMID:25405324; http://dx.doi.org/10.1038/ncomms6490 25405324 PMC4236720

[60] Tao Y, Kassatly RF, Cress WD, Horowitz JM. Subunit composition determines E2F DNA-binding site specificity. Mol Cell Biol 1997; 17:6994-7007; PMID:9372931; http://dx.doi.org/10.1128/MCB.17.12.6994 9372931 PMC232556

[61] Tyagi S, Chabes AL, Wysocka J, Herr W. E2F activation of S phase promoters via association with HCF-1 and the MLL family of histone H3K4 methyltransferases. Mol Cell 2007; 27:107-19; PMID:17612494; http://dx.doi.org/10.1016/j.molcel.2007.05.030 17612494

[62] Landsberg RL, Sero JE, Danielian PS, Yuan TL, Lee EY, Lees JA. The role of E2F4 in adipogenesis is independent of its cell cycle regulatory activity. Proc Natl Acad Sci U S A 2003; 100:2456-61; PMID:12604789; http://dx.doi.org/10.1073/pnas.0138064100 12604789 PMC151362

[63] Lang SE, McMahon SB, Cole MD, Hearing P. E2F transcriptional activation requires TRRAP and GCN5 cofactors. J Biol Chem 2001; 276:32627-34; PMID:11418595; http://dx.doi.org/10.1074/jbc.M102067200 11418595

[64] Magae J, Wu CL, Illenye S, Harlow E, Heintz NH. Nuclear localization of DP and E2F transcription factors by heterodimeric partners and retinoblastoma protein family members. J Cell Sci 1996; 109(Pt 7):1717-26; PMID:8832394 8832394 10.1242/jcs.109.7.1717

[65] Allen KE, de la Luna S, Kerkhoven RM, Bernards R, La Thangue NB. Distinct mechanisms of nuclear accumulation regulate the functional consequence of E2F transcription factors. J Cell Sci 1997; 110(Pt 22):2819-31; PMID:9427290 9427290 10.1242/jcs.110.22.2819

[66] Lindeman GJ, Gaubatz S, Livingston DM, Ginsberg D. The subcellular localization of E2F-4 is cell-cycle dependent. Proc Natl Acad Sci U S A 1997; 94:5095-100; PMID:9144196; http://dx.doi.org/10.1073/pnas.94.10.5095 9144196 PMC24637

[67] Kim J, Woo AJ, Chu J, Snow JW, Fujiwara Y, Kim CG, Cantor AB, Orkin SH. A Myc network accounts for similarities between embryonic stem and cancer cell transcription programs. Cell 2010; 143:313-24; PMID:20946988; http://dx.doi.org/10.1016/j.cell.2010.09.010 20946988 PMC3018841

[68] van Amerongen MJ, Diehl F, Novoyatleva T, Patra C, Engel FB. E2F4 is required for cardiomyocyte proliferation. Cardiovasc Res 2010; 86:92-102; PMID:19955219; http://dx.doi.org/10.1093/cvr/cvp383 19955219

[69] Morillo SM, Abanto EP, Roman MJ, Frade JM. Nerve growth factor-induced cell cycle reentry in newborn neurons is triggered by p38MAPK-dependent E2F4 phosphorylation. Mol Cell Biol 2012; 32:2722-37; PMID:22586272; http://dx.doi.org/10.1128/MCB.00239-12 22586272 PMC3416181

[70] Khidr L, Chen PL. RB, the conductor that orchestrates life, death and differentiation. Oncogene 2006; 25:5210-9; PMID:16936739; http://dx.doi.org/10.1038/sj.onc.1209612 16936739

[71] Sasagawa S, Nishimura Y, Hayakawa Y, Murakami S, Ashikawa Y, Yuge M, Okabe S, Kawaguchi K, Kawase R, Tanaka T. E2F4 Promotes Neuronal Regeneration and Functional Recovery after Spinal Cord Injury in Zebrafish. Front Pharmacol 2016; 7:119; PMID:27242526 27242526 10.3389/fphar.2016.00119 PMC4860404

[72] Scognamiglio R, Cabezas-Wallscheid N, Thier MC, Altamura S, Reyes A, Prendergast AM, Baumgartner D, Carnevalli LS, Atzberger A, Haas S, et al. Myc Depletion Induces a Pluripotent Dormant State Mimicking Diapause. Cell 2016; 164:668-80; PMID:26871632; http://dx.doi.org/10.1016/j.cell.2015.12.033 26871632 PMC4752822

